# Home Monitoring trends during COVID‐19 infection

**DOI:** 10.1002/joa3.12483

**Published:** 2020-12-18

**Authors:** Vincenzo De Simone, Stefania Guardalben, Paola Guarise, Nicola Padovani, Daniele Giacopelli, Gabriele Zanotto

**Affiliations:** ^1^ Cardiology Ospedale Mater Salutis Legnago Italy; ^2^ Clinical unit Biotronik Italia Vimodrone Italy; ^3^ Department of Cardiac, Thoracic, Vascular Sciences & Public Health University of Padova Padua Italy

**Keywords:** COVID‐19, implantable cardioverter defibrillator, pacemaker, remote monitoring, SARS‐CoV‐2

## Abstract

**Background:**

Cardiac implantable electronic device (CIED) recipients could have an unfavorable prognosis if infected with the novel coronavirus (COVID‐19). We aimed to analyze the data daily transmitted by the Home Monitoring (HM) system (BIOTRONIK, Berlin, Germany) of CIEDs during the infection.

**Methods:**

We identified CIED patients followed with the HM who experienced COVID‐19 clinical manifestations. The daily trends of the following HM variables were analyzed: mean heart rate (HR), physical activity, thoracic impedance (TI), ventricular and atrial arrhythmic burden.

**Results:**

The study cohort included 10 CIED patients (median age 90 [84‐92] years, male 90%) with acute respiratory syndrome. The HR showed an increase of a value ranging from 10 to 30 bpm well in advance of the severe clinical manifestations. The physical activity was generally low during the entire infection course. The TI decreased in patients presented with pulmonary edema, but increased significantly (8 to 25 Ω) in most COVID‐19 patients (8 out of 10) suggesting an association with pulmonary fibrosis. Arrhythmic complications were also found in half of the patients.

**Conclusion:**

The trends of HR and TI in CIEDs recipients infected by the COVID‐19 often showed early recurrent patterns before adverse clinical manifestations.

## Graphical abstract

We analyzed longitudinal trends daily transmitted by the Home Monitoring system of CIED patients with confirmed COVID‐19 infection. The heart rate showed the most recurrent pattern with a significant increase well in advance of severe clinical manifestations. As expected, the thoracic impedance decreased in patients presented with pulmonary edema, but increased significantly in most COVID‐19 patients suggesting an association with pulmonary fibrosis. Arrhythmic complications were also found in half of the patients. Continuous Home Monitoring data should be further investigated for early warning of infection as patients with underlying cardiovascular diseases are at high risk of adverse outcomes.

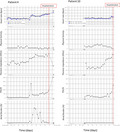

## 1 INTRODUCTION

The pathological manifestation of the novel coronavirus (COVID‐19) infection was reported not only as a viral pneumonia, but also as a complex, multi‐organ systemic disease.^1^, ^2^

The elderly population was the one who experienced the most complicated clinical courses of the infection, with prolonged hospitalizations and high mortality rates.^3^ Among them, cardiac implantable electronic device (CIED) recipients could be even at higher risk due to the presence of underlying cardiovascular diseases.^4^

Remote monitoring (RM) of CIED, which is a well‐established reality in our Cardiology department, automatically provides a wide spectrum of clinical information.^5^ As some of these patients were affected by the COVID‐19 infection, we aimed to explore the transmitted data from their device to investigate potential recurrent and specific patterns of some variables during the infection.

## 2 METHODS

We identified CIED recipients followed with the Home Monitoring (HM) technology (BIOTRONIK) with confirmed COVID‐19 positive testing. Our objective was to analyze the RM transmissions during the infection course and to investigate the association of longitudinal trends of the available clinical variables with COVID‐19‐related adverse events.

The HM technology is a well‐known RM system for CIED patients based on an embedded antenna within the device case which daily transmits stored diagnostic data to a mobile unit connected to a service center for automatic processing and online review. This system is able to transmit technical information for surveillance of device functioning (eg battery status, lead integrity), and clinical information on patient status and arrhythmias occurrence on a daily basis. In this study, we focused on longitudinal trends of the following remotely available clinical variables:

1. Mean heart rate (HR);
2. Physical activity measured with an accelerometer sensor;
3. Thoracic impedance (TI) measured with subthreshold right ventricle‐to‐device can pulses;
4. Frequency of pre‐ventricular contractions (PVCs) per hour;
5. Atrial burden defined as the cumulative time spent in atrial arrhythmia and reported as percentage of 24 hours.

Baseline characteristics, symptoms reported during the period of infection, pharmacological therapy for COVID‐19 treatment and clinical outcome were collected for all patients. Available chest x‐ray images were also reviewed to analyze lungs status.

The longitudinal trends of the HM variables were qualitative analyzed for each patient to detect significant variations before COVID‐19‐related hospitalization, death, or reported symptoms. Each variable trend was then classified as absent, increasing, or decreasing. In case of significant variation, the interval between its onset and the clinical event was defined as the timing of the trend.

Continuous variables were expressed as median (interquartile range) while categorical variables as number (percentage).

## 3 RESULTS

### 3.1 Patients' characteristics and outcome

A total of 10 CIED patients positive for COVID‐19 between February and May 2020 were included in the present analysis. Seven patients were implanted with a pacemaker, two with a cardiac resynchronization therapy defibrillator (CRT‐D), and one with a conventional implantable cardioverter defibrillator (ICD). The study cohort was extremely elderly (median age 90 [84‐92] years) with a high prevalence of male (90%). The majority of patients had known hypertension (80%) and was under loop diuretics (90%).

The most frequent symptoms were fever (90%) and dyspnea (80%). Diuretics (80%), antibiotics (90%), and cortisone (90%) were the most used drugs to treat the infection. A total of nine patients (90%) underwent chest X‐ray during the infection course showing two recurrent pneumonia pictures: pleural effusion and thickening or pleural thickening without effusion. The majority of patients were hospitalized (80%). Overall, 8 (80%) patients died, five from pneumonia, and three from acute worsening heart failure. Seven of them died in hospital, one at home.

### 3.2 Home Monitoring trends

**FIGURE 1:**
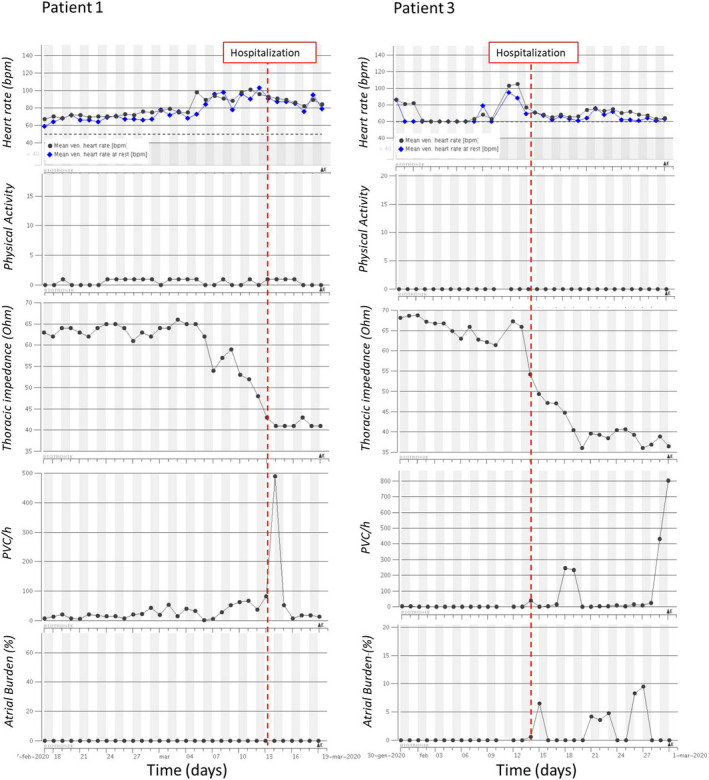
Examples of 1‐month longitudinal trend of Home Monitoring variables (mean and rest heart rate, physical activity, thoracic impedance, number of pre‐ventricular contractions per hour [PVC/h], atrial arrhythmic burden) during COVID‐19 infection for two patients with decreasing trend of thoracic impedance. Red dashed line represents the date of hospitalization

**FIGURE 2:**
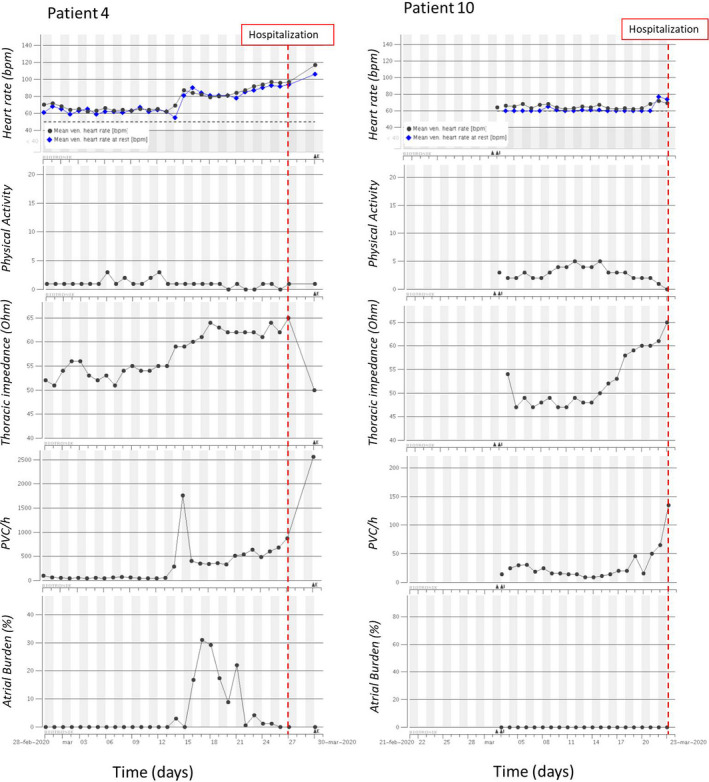
Examples of 1‐month longitudinal trend of Home Monitoring variables (mean and rest heart rate, physical activity, thoracic impedance, number of pre‐ventricular contractions per hour [PVC/h], atrial arrhythmic burden) during COVID‐19 infection for two patients with increasing trend of thoracic impedance. Red dashed line represents the date of hospitalization

Table 1 summarizes patients' outcomes in terms of hospitalization and death, as well as the significant trends of each HM variables observed before COVID‐19 severe manifestations. Figures 1 and 2 depict four examples of 1‐month longitudinal trend of the analyzed HM variables during the infection.

**TABLE 1:** Significant increasing (↗) or decreasing (↘) trends of remote monitoring variables during COVID‐19 infection

Pat	Age	Sex	Device	Death	Hosp	Heart rate		Physical activity		Thoracic impedance		Arrhythmic findings	Chest X‐ray findings
						Trend	Timing	Trend	Timing	Trend	Timing		
1	90	Male	PM D	Yes	Yes	↗	8 days before hosp	Absent	‐	↘	10 days before hosp	PVC	Pleural effusion and thickening
2	92	Male	PM S	Yes	Yes	↗	3 days before hosp	Absent	‐	↗	6 days before hosp	None	Pleural effusion with apical thickening
3	82	Male	CRT‐D	Yes	Yes	↗	3 days before hosp	Absent	‐	↘	3 days before hosp	PVC and new‐onset AF	Pleural effusion and thickening
4	79	Male	CRT‐D	Yes	Yes	↗	11 days before hosp	Absent	‐	↗	11 days before hosp	PVC and new‐onset AF	Pleural thickening without effusion
5	84	Male	ICD D	Yes	Yes	↗	31 days before hosp	↘	10 days before hosp	↗	4 days after hosp	PVC	Pleural thickening without effusion
6	86	Male	PM D	Yes	Yes	n.a.	n.a.	Absent	‐	↗	22 days before hosp	None	Pleural thickening without effusion
7	93	Male	PM S	Yes	No	n.a.	n.a.	Absent	‐	↗	21 days before death	None	‐
8	92	Male	PM S	Yes	Yes	↘	18 days before hosp	Absent	‐	↗	23 days before hosp	None	Pleural thickening without effusion
9	93	Female	PM D	No	No	Absent	‐	↘	2 days before symptoms onset	↗	2 days before symptoms onset	From paroxysmal to persistent AF	Pleural thickening without effusion
10	86	Male	PM D	No	Yes	↗	4 days before hosp	↘	7 days before hosp	↗	8 days before hosp	PVC	Pleural thickening without effusion

Abbreviations: Hosp, hospitalization; PVC, pre‐ventricular contraction; AF, atrial fibrillation; PM D, pacemaker dual‐chamber; PM S, pacemaker single‐chamber; CRT‐D, cardiac resynchronization therapy defibrillator; ICD D, implantable cardioverter defibrillator dual‐chamber; n.a., not available for III°AV block.

#### 3.2.1 Heart rate

The heart rate information was available for eight patients out of 10, as two patients had III° atrioventricular block and 100% ventricular pacing. The mean HR appeared to increase before hospitalization in most cases (6 out of 8). The onset of the increasing trend ranged from few days (Figure 2, Patient 10) to several weeks (Figure 2, Patient 4) before the clinical event. The overall increase in mean HR ranged from 10 to 30 bpm. Interestingly, one patient with a mean HR around 70 bpm showed a decreasing trend starting from 18 days before hospitalization leading to a 100% paced rhythm at 50 bpm (pacemaker basic rate). There were no significant variations in heart rate in only one patient.

#### 3.2.2 Physical activity

The physical activity observed was generally very low due to the patients' advanced age and the lockdown status for the COVID‐19 pandemic. Only three patients out of 10 showed a visible decreasing trend. The activity reduction started from 2 to 10 days prior to the clinical event (Figure 2, Patient 10). All other patients had a very low and flat activity during the entire infection course (Figure 1).

#### 3.2.3 Thoracic impedance

A total of eight patients out of 10 had an increasing pattern of TI with a timing ranging from 2 to 23 days before hospitalization (Figure 2) and an overall increase ranging from 8 to 25 Ω. Two patients presented a decreasing trend starting 3 and 10 days before hospitalization, respectively, with a final absolute reduction of around 25 Ω (Figure 1). Interestingly, all patients with increasing pattern of TI had the pleural thickening without effusion finding at chest x‐ray exam. Only 1 patient with the same pattern of TI showed pleural effusion at the chest x‐ray, but it should be noted that this patient had history of heart failure with an acute worsening reported one month before the COVID‐19 infection. Conversely, in the two patients with decreasing pattern of TI, lungs pleural effusion, and thickening was reported at X‐ray examination.

#### 3.2.4 Arrhythmic findings

A total of 5 patients (50%) had an increasing ventricular ectopic activity during COVID infection. Only in 1 case, the increased frequency of PVCs was observed several days in advance (Figure 2, Patient 4), while in the other cases this variation was observed at the time of hospitalization or some days after (Figure 1, Patient 3).

New‐onset atrial fibrillation (AF) was also observed in 2 cases. Patient 3 had several episodes of AF lasting some hours after the hospital admission (Figure 1), while Patient 4 had AF episodes lasting several hours more than 10 days before the hospitalization (Figure 2). During the infection, one patient presented the progression of AF from a paroxysmal to a persistent form.

## 4 DISCUSSION

In this study, we analyzed the trends of clinical data transmitted by RM of CIEDs in patients infected by the virus COVID 19. The major findings are as follows: (a) in most cases mean HR had an early increasing trend; (b) TI showed two different patterns: increasing in patients without lungs pleural effusion and decreasing in patients presented with pulmonary edema; (c) new‐onset AF and increasing ventricular ectopic activity were observed in several cases.

Among the available variables, the mean HR was the one showing the most recurrent pattern during infection course. A significant increase (from 10 to 30 bpm) was observed in 7 cases out of 8. As this pattern often appeared well in advanced than hospital admission, it could be potentially suggested for early infection screening. This finding was not unexpected as tachycardia is a common feature of sepsis and indicative of a systemic response to stress; it is the physiologic mechanism by which cardiac output, and thus oxygen delivery to tissues, is increased.^6^ Of note, we observed also a single case of drop of mean HR leading to 100% paced rhythm. Transient bradycardia was also reported as possible manifestation of COVID‐19 in a recent case report.^7^

Some interesting observations were also found for the TI. This parameter is measured between the right ventricular lead and the device generator and could detect fluid accumulation in the lungs. The role of TI in predicting a worsening state of compensation has been known for several years; it is inversely correlated with pulmonary capillary wedge pressure and decrease before the onset of patient symptoms for volume overload.^8^ Accordingly, we observed a decreasing trend of TI in two patients (with history of previous congestive heart failure) who then presented at hospital with pulmonary pleural effusion. Conversely, in the majority of patients an increasing trend of this variable was observed and chest X‐ray imaging showed the presence of parenchymal thickening in the absence of pulmonary congestion. It is possible to theorize that TI could increase for the onset of pulmonary fibrosis. Pulmonary fibrotic disease observed in COVID‐19 ranges from fibrosis associated with organizing pneumonia to severe acute lung injury.^9^ Finally, we reported a significant incidence of new‐onset AF and increased ventricular ectopic activity. In our cases, these arrhythmic complications appeared later than the other variables variations to indicate that they could be consequences of the ongoing inflammatory status. Data on arrhythmias during COVID‐19 infection are still scant, but these complications are not unexpected.^10^ According to recent findings, arrhythmias could be also the result of oxidative stress, apoptosis, and fibrosis promoted by inflammation. Thus, it may contribute to both the occurrence/maintenance of AF and its thromboembolic complications.^11^

With the limited number of patients, our results should be considered as preliminary observations to be confirmed in further larger analysis which may also investigate if combinations of HM parameters could predict prognosis in CIED patients with pneumonia related to COVID‐19.

## 5 CONCLUSIONS

The daily trends of mean HR and TI in CIEDs recipients infected with the COVID‐19 virus often showed early recurrent patterns before adverse clinical manifestations. Arrhythmic complications were also observed in some patients in a later stage.

Continuous remote monitoring data could have potential benefits in a context of COVID‐19 pandemic in terms of early screening for more specific diagnostic tests in a high‐risk population.

## CONFLICT OF INTERESTS

D. G. is employee of BIOTRONIK Italia. All the remaining authors declare no Conflict of Interests for this article.
